# Controlled Fabrication of Hierarchically Structured MnO_2_@NiCo-LDH Nanoarrays for Efficient Electrocatalytic Urea Oxidization

**DOI:** 10.3390/nano13152268

**Published:** 2023-08-07

**Authors:** Wenjun Liu, Wenbo Xu, Guofa Dong, Ming Fang

**Affiliations:** 1 Shenzhen Key Laboratory of Special Functional Materials, Guangdong Research Centre for Interfacial Engineering of Functional Materials, College of Materials Science and Engineering, Shenzhen University, Shenzhen 518060, China; liuwj@szu.edu.cn (W.L.); xwbxwbxwb1996@gmail.com (W.X.); 2 Fujian Key Laboratory of Functional Marine Sensing Materials, College of Materials and Chemical Engineering, Minjiang University, Fuzhou 350108, China

**Keywords:** urea oxidization, hierarchical nanostructure, nickel cobalt hydroxide

## Abstract

Urea, a prevalent component found in wastewater, shows great promise as a substrate for energy-efficient hydrogen production by electrolysis. However, the slow kinetics of the anodic urea oxidation reaction (UOR) significantly hamper the overall reaction rate. This study presents the design and controlled fabrication of hierarchically structured nanomaterials as potential catalysts for UOR. The prepared MnO_2_@NiCo-LDH hybrid catalyst demonstrates remarkable improvements in reaction kinetics, benefiting from synergistic enhancements in charge transfer and efficient mass transport facilitated by its unique hierarchical architecture. Notably, the catalyst exhibits an exceptionally low onset potential of 1.228 V and requires only 1.326 V to achieve an impressive current density of 100 mA cm^−2^, representing a state-of-the-art performance in UORs. These findings highlight the tremendous potential of this innovative material designing strategy to drive advancements in electrocatalytic processes.

## 1. Introduction

As a promising and environmentally-friendly energy carrier, hydrogen (H_2_) has garnered significant attention as a potential substitute for fossil fuels in future energy systems [1,2]. The electrochemical water splitting reaction (2H_2_O → 2H_2_ + O_2_) provides an eco-friendly and sustainable pathway for hydrogen production, enabling the efficient conversion of surplus or low-quality electricity into chemical energy stored in the form of hydrogen gas [3,4,5,6]. Regrettably, the efficiency of water electrolysis is significantly impeded due to the high theoretical reaction potential of 1.23 V and the sluggish kinetics of the oxygen evolution reaction (OER) occurring at the anode side [7]. In order to address this limitation, several novel reaction schemes have been proposed as alternatives to the OER. The urea oxidation reaction (UOR, CO(NH_2_)_2_ + 6OH^−^ → N_2_ + 5H_2_O + CO_2_ + 6e^−^), with a favorable thermodynamic potential of only 0.37 V, has therefore attracted considerable research interest [8]. Urea, which occurs in abundance in human urine and/or industrial wastewater, serves as an resource for this reaction. Despite its great promise, the UOR still encounters significant kinetic barriers that hinder its efficiency [9,10]. Therefore, the development of active catalysts is imperative to overcome these challenges. Numerous transition metal compounds have been extensively exploited as potential catalysts for the UOR [10,11,12,13,14,15,16,17,18]. Among them, Ni–Co binary hydroxides stand out as particularly attractive options due to their abundant availability in nature and remarkable catalytic performance for UORs [17,19,20]. Despite great promise, there remains a considerable requirement for further enhancing performance to meet the demands of practical applications, calling for the implementation of innovative material design strategies. Catalytic reactions take place at the catalyst’s surface, which underscores the importance of reducing its size to maximize the availability of active sites. However, size reduction often results in increased boundaries that impede electron conduction. To strike a balance, the implementation of hierarchical 3D nanostructures arises as the superior choice, as they can offer exceptionally large surface areas while preserving well-connected networks for effective charge carrier conduction.

In this article, we present the design and fabrication of hierarchically structured MnO_2_@NiCo-LDH nanosheet arrays supported on nickel foam (NF) and their applications as an efficient UOR electrocatalyst. Ultrathin MnO_2_ nanosheet arrays were first formed on NF via hydrothermal growth, which served as a conductive substrate for the electrodeposition of a (Ni, Co)-based layered double hydroxide (NiCo-LDH) nanosheet. The NiCo-LDH loading density was readily controlled by tuning the deposition current density and time. The formation of the hierarchical nanostructure was confirmed by microscopic investigations, and remarkable enhancement in electrocatalytic performance for UOR was validated by leveraging the advantages of its unique architecture.

## 2. Materials and Methods

### 2.1. Preparation of MnO_2_@NiCo-LDH Nanosheet Arrays

Commercial nickel foam (NF, 1 mm in thickness) was cut into pieces measuring 1 cm × 3 cm for subsequent use. These nickel foam pieces underwent an initial treatment in a 1 M hydrochloric acid solution for 15 min to remove the naturally formed oxide layer at the surface.

The cleaned NF pieces were utilized as substrates for the growth of ultrathin MnO_2_ nanosheet arrays via hydrothermal synthesis by adopting our previously reported method [21]. Typically, a piece of cleaned NF was placed in a 50 mL polytetrafluoroethylene (PTFE)-lined stainless steel reaction vessel that was loaded with 30 mL of 50 mM aqueous solution of KMnO_4_, and the reaction was then held at 180 °C for 2.5 h in an electric oven. Electrodeposition was employed to form NiCo-LDH nanosheets over the primary MnO_2_ nanosheet arrays from aqueous solutions of the metal nitrate salts, with the MnO_2_-coated nickel foam as the working electrode, a piece of platinum (Pt) foil as the counter electrode, and a saturated calomel electrode (SCE) as the reference electrode. The electrolyte was an aqueous solution that contained 0.1 M Ni(NO_3_)_2_·6H_2_O and 0.1 M Co(NO_3_)_2_·6H_2_O. Typically, the deposition was carried out at a constant current density of −20 mA cm^−2^, and the NiCo-LDH loading amount was controlled by tuning the deposition time (50 s, 100 s, 150 s, 200 s).

### 2.2. Characterizations

Scanning electron microscopy (SEM) was acquired in a secondary electron collection mode with an accelerating voltage of 5 KeV by using an APREO-S field emission scanning electron microscope (Thermo Fisher Scientific Inc., Waltham, MA, USA). Transmission electron microscopy (TEM) investigations were conducted on an accelerating voltage of 200 KeV on the JEM-F200 scanning transmission electron microscope (JEOL Ltd., Akishima, Tokyo, Japan). A ESCALAB X-ray photoelectron spectrometer (Thermo Fisher Scientific Inc., Waltham, MA, USA) was used for collecting X-ray photoelectron spectroscopy (XPS) data and all spectra were calibrated by aligning the C 1s peak at 284.8 eV. X-ray diffraction (XRD) patterns were obtained using a RIGAKU Miniflex X-ray diffractometer (Applied Rigaku Technologies, Inc., Akishima, Tokyo, Japan), with Cu-Kα radiation, at a scan rate of 5° per minute. Inductively coupled plasma-atomic emission spectroscopy (ICP-AES) analysis was performed on an iCAP 7000 Series spectrometer (Thermo Fisher Scientific Inc., Waltham, MA, USA) to assess the possible dissolution of the catalyst component after the prolonged electrochemical stability test. For the analysis, 2 mL of the electrolyte was taken and then acidified using 4 mL of 1 M HCl. The solution was further filtered through a 0.22 μm nylon filter before being sent for the ICP-AES test.

### 2.3. Electrochemical Measurements

All electrochemical measurements were performed by using a Interface 1010E potentiostat (Gamry Instruments, Warminster, PA, USA). The OER tests were carried out in a standard 1 M KOH (pH = 13.6) solution. For the UOR tests, 0.5 M urea was added to the alkaline solution. Linear sweep voltammetry (LSV) curves were recorded with a scan rate of 5 mV/s, and iR-correction was applied using the current interrupt (CI) method. Electrochemical impedance spectroscopy (EIS) measurements were conducted in the potential static mode, using frequencies ranging from 1 to 10^5^ Hz and an oscillation amplitude of 10 mV at an overpotential of 450 mV vs. Hg/HgO (balanced with 1 M KOH). The stability of the electrocatalyst was tested using chronopotentiometry at a constant current density of 100 mA cm^−2^ for a duration of 20 hours. To account for the pH effects, the potentials were converted to the reversible hydrogen electrode (RHE) scale using the Nernst equation (E vs. RHE = E vs. ref + 0.059 × pH + 0.114 V). The potential of the Hg/HgO electrode was also calibrated against a Pt|H_2_ (g) electrode in the 1 M KOH electrolyte using a multimeter [21], which showed a potential of 0.916 V (Figure S1), consistent with the calculation mentioned above.

## 3. Results and Discussion

Ultrathin MnO_2_ nanosheet arrays supported on NF were utilized as a substrate for the deposition of NiCo-LDH. These MnO_2_ nanosheet arrays were selected because of their half-metallic electronic properties, easy synthesis, and large accessible surface areas [22,23]. SEM images (Figure S2) confirm the formation of vertically aligned MnO_2_ nanosheets on the surface of NF, which was consistent with our previous report [21]. The bare NF and NF-supported MnO_2_ nanosheet arrays were employed as substrates for the electrodeposition of the NiCo-LDH active layer from solutions containing nickel and cobalt nitrate salts. As depicted in Equations (1) and (2), the deposition was triggered through an increase in local pH at the electrode’s surface as a result of the electrochemical reduction in nitrate ions [24].

(1) $${\mathrm{NO}}_{3}^{-}+7{\;H}_{2}O+8\;e^{-}\to {\mathrm{NH}}_{4}^{+}+10{\;\mathrm{OH}}^{-}$$

(2) $$x{\;\mathrm{Ni}}^{2+}+y{\;\mathrm{Co}}^{2+}+2\left(x+y\right){\;\mathrm{OH}}^{-}\to {\mathrm{Ni}}_{x}{\mathrm{Co}}_{y}{\left(\mathrm{OH}\right)}_{2}$$

**Figure 1:**
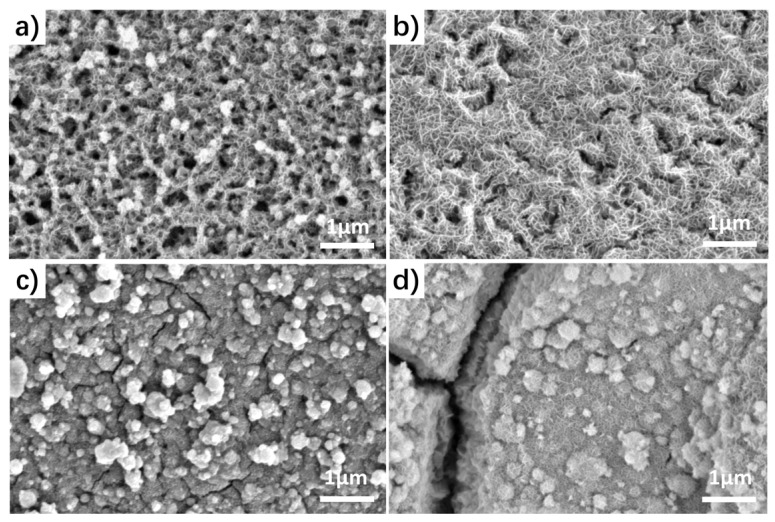
SEM images of MnO_2_@NiCo-LDH with electrodeposition times of (**a**) 50 s, (**b**) 100 s, (**c**) 150 s, and (**d**) 200 s.

The formation of NiCo-LDH over the MnO_2_ was investigated using SEM. As shown in Figure 1, when the deposition was carried out at the current density of −20 mA cm^−2^ and kept for a short time of 50 s, a uniform coating of LDH nanosheets was observed on the surfaces of individual MnO_2_ nanosheets. Importantly, there were still ample free spaces present among the MnO_2_@NiCo-LDH hybrid nanosheets. However, as the deposition time increased, a continuous overcoating of LDH occurred. For example, after deposition for 150 s, nearly all the available spaces between the primary MnO_2_ nanosheets were occupied with LDH, and concurrently, cracks started to form within the overall deposition layer. These cracks became more pronounced as the deposition time was further extended to 200 s. As a control, NiCo-LDH was also deposited on bare NF, and the SEM image reveals that the LDH nanosheets were organized into close-packed microspheres (Figure S3).

**Figure 2:**
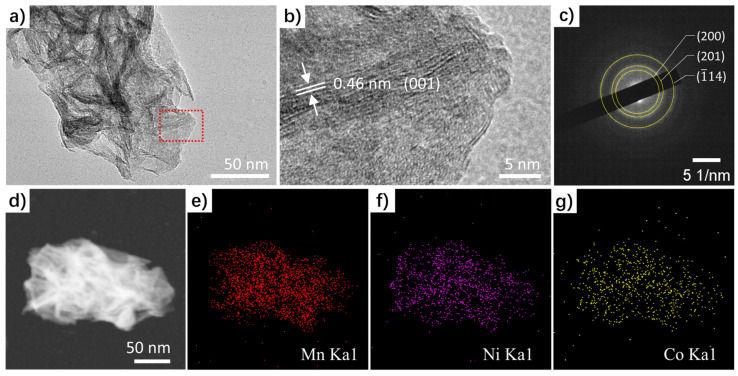
(**a**) TEM, (**b**) HRTEM, (**c**) SAED pattern, and (**d**–**g**) HAADF STEM images and the corresponding EDS elemental mapping images of MnO_2_@NiCo-LDH.

With a hierarchical structure, the MnO_2_@NiCo-LDH fabricated with a deposition time of 50 s was further investigated in TEM. The presence of vertically aligned NiCo-LDH over the MnO_2_ sheets was clearly demonstrated by the emergence of conspicuous black strips observed atop the bulk nanosheets in the TEM image (Figure 2a). Upon conducting further analysis using high-resolution transmission electron microscopy (HRTEM), distinct lattice fringes were observed, with a measured spacing of 0.46 nm (Figure 2b). These fringes are indicative of the interlayer spacing of the (001) atomic planes of the hydroxide, as documented in the hydroxide’s crystallographic database entry (PDF#73-1520). Notably, the selected area electron diffraction (SAED) pattern (Figure 2c) exhibited exclusive diffraction rings that matched the δ-MnO_2_ phase (PDF#80-1098), with no indication of NiCo-LDH signals observed, suggesting the low crystallinity of the hydroxide deposits. Moreover, the high-angle annular dark field (HAADF) scanning transmission electron (STEM) image, combined with the energy-dispersive X-ray spectroscopy (EDS) mapping images (Figure 2d–g), provided compelling evidence of the homogeneous distribution of the Mn, Ni, and Co components throughout the sample. The general atomic contents of these elements were determined to be 16.41% (Mn), 7.13% (Ni), and 4.81% (Co), according to the EDS spectrum (Figure S4).

**Figure 3:**
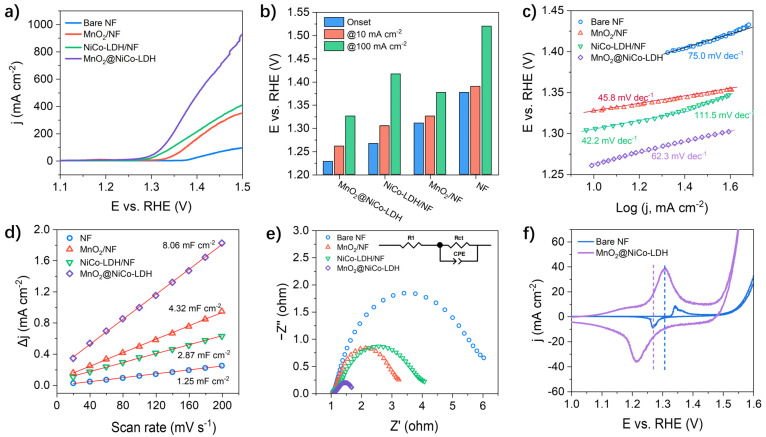
(**a**) LSV curves, (**b**) performance statistics, (**c**) Tafel curves, (**d**) double layer capacitance calculation, (**e**) EIS spectra of MnO_2_@NiCo-LDH and the references of NiCo-LDH/NF, MnO_2_/NF, and NF tested in a 1 M KOH solution added with 0.5 M urea. The inset shows the equivalent circuit of the EIS data. Note: the R_1_, R_ct_, and CPE represent the series resistance, charge transfer resistance, and constant phase element, respectively. (**f**) CV curves of MnO_2_@NiCo-LDH and NF tested in 1 M KOH.

The performance of MnO_2_@NiCo-LDH for urea oxidation reactions (UOR) was assessed in the alkaline solution added with urea (0.5 M). NiCo-LDH/NF, MnO_2_/NF, and bare NF were used as reference catalysts. The LSV curves and potential statistics required to achieve specified current densities are presented in Figure 3a,b, respectively. It is evident that MnO_2_@NiCo-LDH possesses a significantly improved performance for UORs compared to the reference catalysts. Notably, by enlarging the low current density range of the LSV curves (Figure S5), the onset potential of MnO_2_@NiCo-LDH was measured as 1.228 V vs. RHE. This value is below both the thermodynamic redox potential of the OER (1.230 V) and the onset potentials of NiCo-LDH/NF (1.267 V), MnO_2_/NF (1.311 V), and NF (1.377 V). Significantly, when subjected to an applied potential of 1.5 V vs. RHE, the current density of MnO_2_@NiCo-LDH impressively reaches 905 mA cm^−2^. This value surpasses the current densities of NiCo-LDH/NF (398 mA cm^−2^) and MnO_2_/NF (337 mA cm^−2^) by more than two-fold. Likewise, to attain current densities of 10 and 100 mA cm^−2^, the MnO_2_@NiCo-LDH necessitates potentials of 1.261 and 1.326 V vs. RHE, respectively. These values represent a considerable reduction compared to the potentials required by the reference catalysts. Furthermore, they are on par with the most superior UOR catalysts reported in the literature [20] (Table S1), underscoring the exceptional electrochemical capability of MnO_2_@NiCo-LDH. In addition, Tafel plots (Figure 3c) were generated based on the LSV curves for analyzing the kinetics of the UOR on the different catalysts. MnO_2_/NF displayed a Tafel slope of 45.8 mV dec^−1^, indicating a rapid UOR rate. However, its overall performance was limited due to a high onset potential. On the other hand, NiCo-LDH/NF exhibited a Tafel slope of only 42.2 mV dec^−1^ in the low potential region, but this slope significantly increased to 111.5 mV dec^−1^ at higher applied potentials. This discontinuity is likely attributed to an increase in mass transport resistance caused by an insufficient exposed surface area at high potentials. In comparison to the aforementioned catalysts, MnO_2_@NiCo-LDH demonstrated a larger Tafel slope of 62.3 mV dec^−1^, but this value remained consistent across the displayed potential range. These findings corroborate our hypothesis that the combination of high conductivity in MnO_2_ nanosheets and the low onset potential of NiCo-LDH work synergistically to enhance the activity of the hybrid catalyst. The electrochemical surface areas (ECSA) of the catalysts were estimated by measuring their double-layer capacitance (C_dl_) at the non-faradaic region through CV scanning at different rates. The definitive C_dl_ values were calculated as half the slopes of the plots that depict the difference in current densities (Δj) between the anodic and cathodic scans against the scanning rate (Figure 3d and Figure S6). The C_dl_ of MnO_2_@NiCo-LDH reaches 4.03 mF cm^−2^, which is almost twice that of MnO_2_/NF (2.16 mF cm^−2^) and 2.8 times that of NiCo-LDH (1.44 mF cm^−2^). This significant increase in C_dl_ of MnO_2_@NiCo-LDH reflects a much greater accessible surface area for the electrocatalytic reaction. EIS was utilized to further explore the charge transport kinetics of the UOR. As shown in Figure 3e, all the samples exhibit typical semicircular curves in their EIS, where the diameters of the semicircles reflect the charge transfer resistances (R_ct_). MnO_2_@NiCo-LDH has the smallest semicircle diameter, indicating a lowered R_ct_ at the catalyst/electrolyte interface. In earlier investigations, Botte et al. [25,26] suggested an indirect “electrochemical-chemical” (E-C) mechanism for the UOR process on nickel-based catalysts. Based on this theory, electrochemically generated Ni^3+^ ions act as the active species responsible for the oxidation of adsorbed urea molecules. Therefore, the potential for the regeneration of Ni^2+^ to Ni^3+^ plays a critical role in the catalytic process. Figure 3f illustrates the cyclic voltammetry (CV) curves of MnO_2_@NiCo-LDH and bare NF, revealing that the redox potential of Ni^2+^/Ni^3+^ in MnO_2_@NiCo-LDH is shifted to a lower value compared to the pure nickel-based sample. This shift indicates that urea oxidation on MnO_2_@NiCo-LDH can occur at a lower onset potential, consistent with the results obtained from the LSV curves of UOR.

**Figure 4:**
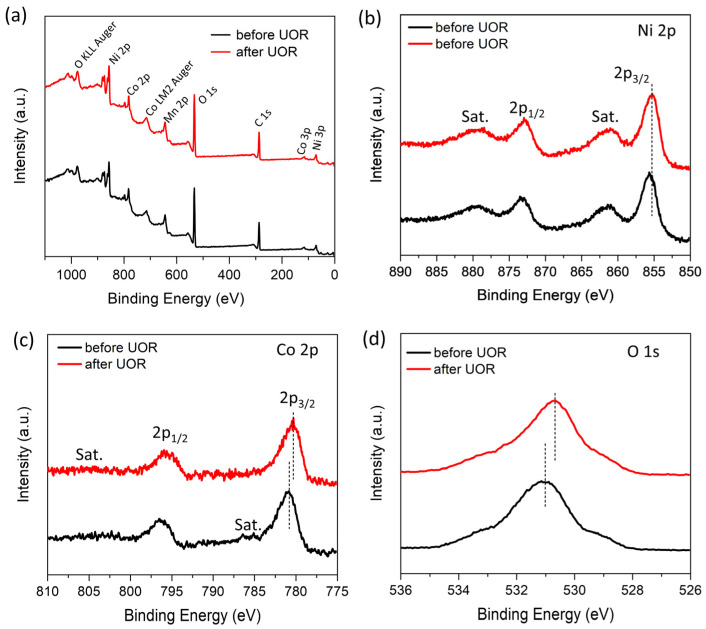
(**a**) Full XPS survey spectra, (**b**–**d**) high-resolution XPS spectra of Ni 2p, Co 2p and O 1s of the MnO_2_@NiCo-LDH before and after UOR tests.

XPS was utilized to investigate any potential alteration in the chemical states of the Ni and Co components during the electrolysis. Figure 4 shows the full XPS survey spectra and the high-resolution spectra of the Ni 2p, Co 2p, and O 1s of the sample before and after the UOR test, all of which were calibrated by aligning the C-C binding energy to the standard value of 284.8 eV in the high-resolution spectra of C 1s (Figure S7). The full survey spectra (Figure 4a) showed no significant change observed in the composition. In the high resolution spectra of Ni 2p (Figure 4b), the initial sample exhibited typical features of 2p_3/2_ and 2p_1/2_, and two satellites at binding energies of 855.4, 873.3 eV, 861.5, and 879.3 eV, signifying that the Ni had a valence state of +2 [27]. Interestingly, after the UOR test, the Ni 2p spectra exhibited no noticeable position shift, indicating little change of the Ni valence. In the spectra of Co 2p (Figure 4c) of the original sample, the photoemission peak of the 2p_3/2_ components was located at 780.8 eV with noticeable satellite peaks emerging at around 785.5 eV, which has been typically observed in Co(OH)_2_ [28]; however, after the UOR test, the 2p_3/2_ peak shifted to a lower binding energy of 780.3 eV, and meanwhile, the satellite peaks were depleted, indicating the formation of Co^3+^ species by the UOR test [29,30]. The O 1s spectrum (Figure 4d) of MnO_2_@NiCo-LDH demonstrated a dominate peak at 510.0 eV, which was ascribed to the oxygen in the hydroxide group (OH^−^) [31]. The peak shifted slightly to the binding energy centered at 530.6 eV after the UOR test, which was likely associated with the absorbed carbonate anions at the surface due to the generation of CO_2_ during UOR. During UOR tests at the high potential region, Ni^2+^ and Co^2+^ ions undergo partial oxidation, forming Ni^3+^ and Co^3+^ species, respectively. However, the Ni 2p spectrum of the sample after UOR revealed the absence of Ni^3+^, indicating the rapid backward reduction of these high-valency species to Ni^2+^. The results obtained provide compelling evidence in favor of the indirect electrochemical (E-C) mechanism of UOR, where the electrochemically generated Ni^3+^ species undergo a simultaneous reaction with urea molecules, leading to their reduction back to Ni^2+^. The regenerated Ni^2+^ species can actively partake in subsequent cycles of the electrocatalysis process.

The catalysts obtained with extended deposition times (100 s, 150 s, 200 s) were also evaluated for their performances in the urea oxidation reactions (UOR). However, their performances were found to be subpar compared to the sample deposited for 50 s, particularly in the high-potential regions (Figure S8). This outcome is to be expected due to the presence of additional NiCo-LDH over-coatings, which exhibit poor conductivity and consequently impede the efficient transfer of electrons from the supporting electrode to the catalytic sites. Furthermore, these over-coatings obstruct the available space between the nanosheets, thereby impeding the mass-transport processes at the surfaces of the electrodes.

The above analyses strongly support the idea that the strategic integration of MnO_2_ nanosheets and NiCo-LDH within a hierarchical architecture not only amplifies the exposure and accessibility of active sites from the NiCo-LDH components but also promotes effective electron and reactant transfer during the electrochemical process, and this remarkable synergy leads to a state-of-the-art performance for UOR.

To showcase the advantages of UOR as an alternative anodic reaction to OER, a comparison was made by measuring the LSV curve of MnO_2_@NiCo-LDH (deposited for 50 s) in a pure KOH solution (1 M). As illustrated in Figure S9, the LSV curves of UOR emerge at a much lower potential region, showing a negative shift (ΔE) of 274 mV, compared to that of OER (1.60 V vs. RHE) for achieving a current density of 100 mA cm^−2^, which corresponds to a 17.1% reduction in energy consumption by replacing the OER with UOR. It is noteworthy that the shift is much larger than that of NiCoGe oxyhydroxide (260 mV), the best reported catalyst of UOR [20]. Considering that the best reported OER catalysts typically require an overpotential of about 250 mV (or a potential of 1.480 V vs. RHE) to achieve the same current level [32], the energy saved still amounts to 10.4%. This finding suggests great promise in adopting UOR as a replacement anodic reaction in energy-saving hydrogen production.

**Figure 5:**
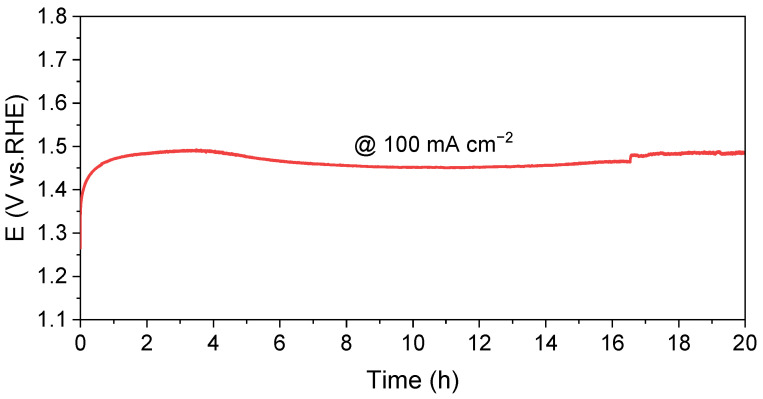
Chronopotential curve of MnO_2_@NiCo-LDH at 100 mA cm^−2^.

A constant-current chronopotentiometry measurement was carried out to evaluate the stability of the catalyst. Figure 5 illustrates the potential recorded over a time course of 20 h at a current density of 100 mA cm^−2^. It is evident that the potential experienced only a minor change during the time course, demonstrating the excellent catalytic stability of MnO_2_@NiCo-LDH for UOR. Additionally, an ICP-AES was performed to analyze the possible presence of dissolved metal cations resulting from the effect of urea complexation. The concentrations of dissolved Co, Ni, and Mn cations were found to be 0.180 ppm, 0.045 ppm, and 0.001 ppm, respectively. These remarkably low values provide further evidence of the outstanding chemical stability of the electrocatalysts under the operational conditions.

## 4. Conclusions

In summary, this study focused on the development of a precisely engineered hierarchical structure of MnO_2_@NiCo-LDH nanoarrays for effective electrocatalytic urea oxidation. The synthesized hybrid catalyst showcased improved reaction kinetics, which can be attributed to the combined advantages of the high electrical conductivity of MnO_2_ nanosheets and the intrinsic activity of the NiCo-LDH deposits. Additionally, the three-dimensional hierarchical architecture provided ample space for efficient mass transport during the reaction. Remarkably, the resulting catalyst exhibited an onset potential of only 1.228 V for UOR and required only 1.326 V to achieve a current density of 100 mA cm^−2^. Overall, the controlled fabrication of hierarchically structured nanoarrays offers a potential pathway for efficient electrocatalytic urea oxidation and holds great promise for application in energy conversion and storage systems.

## Data Availability

Data are available on request from the authors.

## Supplementary Materials

The following supporting information can be downloaded at: https://www.mdpi.com/article/10.3390/nano13152268/s1, Figure S1: Potential calibration of the SCE and Hg/HgO against a Pt|H_2_(g) electrode in 1 M KOH; Figure S2: SEM images of MnO_2_ nanosheet arrays grown on nickel foam (NF); Figure S3: SEM image of CoNi-LDH deposited on NF; Figure S4: EDS spectrum of the mapping area; Figure S5: Enlarged view of the UOR LSV curves of different catalysts; Figure S6: CV curves of (a) MnO_2_@NiCo-LDH, (b) NiCo-LDH/NF, (c) MnO_2_/NF, and (d) NF at different potential scan rates at the non-Faradaic region for ECSA estimations; Figure S7: High resolution calibrated C 1s XPS spectra of MnO_2_@NiCo-LDH before and after the UOR test; Figure S8: LSV curves of UOR by MnO_2_@NiCo-LDH with different deposition times; Figure S9: LSV curves of MnO_2_@NiCo-LDH deposited for 50 s for UOR and OER. Table S1: Performance comparison of UOR catalysts reported recently.

## References

[1] Yue M., Lambert H., Pahon E., Roche R., Jemei S., Hissel D. (2021). Hydrogen Energy Systems: A Critical Review of Technologies, Applications, Trends and Challenges. Renew. Sustain. Energy Rev..

[2] Yusaf T., Laimon M., Alrefae W., Kadirgama K., Dhahad H.A., Ramasamy D., Kamarulzaman M.K., Yousif B. (2022). Hydrogen Energy Demand Growth Prediction and Assessment (2021–2050) Using a System Thinking and System Dynamics Approach. Appl. Sci..

[3] Shih A.J., Monteiro M.C.O., Dattila F., Pavesi D., Philips M., da Silva A.H.M., Vos R.E., Ojha K., Park S., van der Heijden O. (2022). Water Electrolysis. Nat. Rev. Methods Primer.

[4] Yu Z.-Y., Duan Y., Feng X.-Y., Yu X., Gao M.-R., Yu S.-H. (2021). Clean and Affordable Hydrogen Fuel from Alkaline Water Splitting: Past, Recent Progress, and Future Prospects. Adv. Mater..

[5] Miller H.A., Bouzek K., Hnat J., Loos S., Bernäcker C.I., Weißgärber T., Röntzsch L., Meier-Haack J. (2020). Green Hydrogen from Anion Exchange Membrane Water Electrolysis: A Review of Recent Developments in Critical Materials and Operating Conditions. Sustain. Energy Fuels.

[6] Qi J., Zhang W., Cao R. (2018). Solar-to-Hydrogen Energy Conversion Based on Water Splitting. Adv. Energy Mater..

[7] Chatenet M., Pollet B.G., Dekel D.R., Dionigi F., Deseure J., Millet P., Braatz R.D., Bazant M.Z., Eikerling M., Staffell I. (2022). Water Electrolysis: From Textbook Knowledge to the Latest Scientific Strategies and Industrial Developments. Chem. Soc. Rev..

[8] Xiao Z., Qian Y., Tan T., Lu H., Liu C., Wang B., Zhang Q., Sarwar M.T., Gao R., Tang A. (2022). Energy-Saving Hydrogen Production by Water Splitting Coupling Urea Decomposition and Oxidation Reactions. J. Mater. Chem. A.

[9] Geng S.-K., Zheng Y., Li S.-Q., Su H., Zhao X., Hu J., Shu H.-B., Jaroniec M., Chen P., Liu Q.-H. (2021). Nickel Ferrocyanide as a High-Performance Urea Oxidation Electrocatalyst. Nat. Energy.

[10] Qin H., Ye Y., Li J., Jia W., Zheng S., Cao X., Lin G., Jiao L. (2022). Synergistic Engineering of Doping and Vacancy in Ni(OH)_2_ to Boost Urea Electrooxidation. Adv. Funct. Mater..

[11] Ji Z., Song Y., Zhao S., Li Y., Liu J., Hu W. (2022). Pathway Manipulation via Ni, Co, and V Ternary Synergism to Realize High Efficiency for Urea Electrocatalytic Oxidation. ACS Catal..

[12] Cai M., Zhu Q., Wang X., Shao Z., Yao L., Zeng H., Wu X., Chen J., Huang K., Feng S. (2023). Formation and Stabilization of NiOOH by Introducing α-FeOOH in LDH: Composite Electrocatalyst for Oxygen Evolution and Urea Oxidation Reactions. Adv. Mater..

[13] Sun H., Liu J., Kim H., Song S., Fei L., Hu Z., Lin H.-J., Chen C.-T., Ciucci F., Jung W. (2022). Ni-Doped CuO Nanoarrays Activate Urea Adsorption and Stabilizes Reaction Intermediates to Achieve High-Performance Urea Oxidation Catalysts. Adv. Sci..

[14] Fang M., Xu W.-B., Han S., Cao P., Xu W., Zhu D., Lu Y., Liu W. (2021). Enhanced Urea Oxidization Electrocatalysis on Spinel Cobalt Oxide Nanowires via on-Site Electrochemical Defect Engineering. Mater. Chem. Front..

[15] Fang M., Xu W.-B., Shen Y., Cao P., Han S., Xu W., Zhu D., Lu Y., Liu W. (2021). Electrochemical Tuning of Nickel Molybdate Nanorod Arrays towards Promoted Electrocatalytic Urea Oxidization. Appl. Catal. Gen..

[16] Tong Y., Chen P., Zhang M., Zhou T., Zhang L., Chu W., Wu C., Xie Y. (2018). Oxygen Vacancies Confined in Nickel Molybdenum Oxide Porous Nanosheets for Promoted Electrocatalytic Urea Oxidation. ACS Catal..

[17] Wang X.-H., Hong Q.-L., Zhang Z.-N., Ge Z.-X., Zhai Q.-G., Jiang Y.-C., Chen Y., Li S.-N. (2022). Two-Dimensional Nickel–Cobalt Bimetallic Hydroxides towards Urea Electrooxidation. Appl. Surf. Sci..

[18] Li D., Zhou X., Liu L., Ruan Q., Zhang X., Wang B., Xiong F., Huang C., Chu P.K. (2023). Reduced Anodic Energy Depletion in Electrolysis by Urea and Water Co-Oxidization on NiFe-LDH: Activity Origin and Plasma Functionalized Strategy. Appl. Catal. B Environ..

[19] Song W., Xu M., Teng X., Niu Y., Gong S., Liu X., He X., Chen Z. (2021). Construction of Self-Supporting, Hierarchically Structured Caterpillar-like NiCo_2_S_4_ Arrays as an Efficient Trifunctional Electrocatalyst for Water and Urea Electrolysis. Nanoscale.

[20] Wang P., Bai X., Jin H., Gao X., Davey K., Zheng Y., Jiao Y., Qiao S.-Z. (2023). Directed Urea-to-Nitrite Electrooxidation via Tuning Intermediate Adsorption on Co, Ge Co-Doped Ni Sites. Adv. Funct. Mater..

[21] Fang M., Han D., Xu W.-B., Shen Y., Lu Y., Cao P., Han S., Xu W., Zhu D., Liu W. (2020). Surface-Guided Formation of Amorphous Mixed-Metal Oxyhydroxides on Ultrathin MnO_2_ Nanosheet Arrays for Efficient Electrocatalytic Oxygen Evolution. Adv. Energy Mater..

[22] Wang H., Zhang J., Hang X., Zhang X., Xie J., Pan B., Xie Y. (2015). Half-Metallicity in Single-Layered Manganese Dioxide Nanosheets by Defect Engineering. Angew. Chem. Int. Ed..

[23] Zhao Y., Chang C., Teng F., Zhao Y., Chen G., Shi R., Waterhouse G.I.N., Huang W., Zhang T. (2017). Defect-Engineered Ultrathin δ-MnO_2_ Nanosheet Arrays as Bifunctional Electrodes for Efficient Overall Water Splitting. Adv. Energy Mater..

[24] Li Z., Shao M., An H., Wang Z., Xu S., Wei M., Evans D.G., Duan X. (2015). Fast Electrosynthesis of Fe-Containing Layered Double Hydroxide Arrays toward Highly Efficient Electrocatalytic Oxidation Reactions. Chem. Sci..

[25] Vedharathinam V., Botte G.G. (2013). Direct Evidence of the Mechanism for the Electro-Oxidation of Urea on Ni(OH)_2_ Catalyst in Alkaline Medium. Electrochim. Acta.

[26] Vedharathinam V., Botte G.G. (2012). Understanding the Electro-Catalytic Oxidation Mechanism of Urea on Nickel Electrodes in Alkaline Medium. Electrochim. Acta.

[27] Biesinger M.C., Payne B.P., Grosvenor A.P., Lau L.W.M., Gerson A.R., Smart R.S.C. (2011). Resolving Surface Chemical States in XPS Analysis of First Row Transition Metals, Oxides and Hydroxides: Cr, Mn, Fe, Co and Ni. Appl. Surf. Sci..

[28] Xu Y., Liu Z., Chen D., Song Y., Wang R. (2017). Synthesis and Electrochemical Properties of Porous α-Co(OH)_2_ and Co_3_O_4_ Microspheres. Prog. Nat. Sci. Mater. Int..

[29] Liardet L., Hu X. (2018). Amorphous Cobalt Vanadium Oxide as a Highly Active Electrocatalyst for Oxygen Evolution. ACS Catal..

[30] Ye S., Wang J., Hu J., Chen Z., Zheng L., Fu Y., Lei Y., Ren X., He C., Zhang Q. (2021). Electrochemical Construction of Low-Crystalline CoOOH Nanosheets with Short-Range Ordered Grains to Improve Oxygen Evolution Activity. ACS Catal..

[31] Zhang H., Tian W., Guo X., Zhou L., Sun H., Tadé M.O., Wang S. (2016). Flower-like Cobalt Hydroxide/Oxide on Graphitic Carbon Nitride for Visible-Light-Driven Water Oxidation. ACS Appl. Mater. Interfaces.

[32] Chowdhury P.R., Medhi H., Bhattacharyya K.G., Hussain C.M. (2023). Recent Progress in the Design and Functionalization Strategies of Transition Metal-Based Layered Double Hydroxides for Enhanced Oxygen Evolution Reaction: A Critical Review. Coord. Chem. Rev..

