# Supplementary material for: Controlled Fabrication of Hierarchically Structured MnO2@NiCo-LDH Nanoarrays for Efficient Electrocatalytic Urea Oxidization
Source: Nanomaterials (Basel). 2023 Aug 7;13(15):2268. doi: 10.3390/nano13152268 (PMC10421065; doi:10.3390/nano13152268)
Supplement: Supplementary file 1 [file nanomaterials-13-02268-s001.zip › nanomaterials-2511047-supplementary.pdf]

Supplementary data for

Controlled Fabrication of Hierarchically Structured MnO_2@NiCo-LDH Nanoarrays for Efficient Electrocatalytic Urea Oxidization

Wenjun Liu [1], Wenbo Xu [1], Guofa Dong [2,*] and Ming Fang [1,*]

[1] Shenzhen Key Laboratory of Special Functional Materials, Guangdong Research Centre for Interfacial Engineering of Functional Materials, College of Materials Science and Engineering, Shenzhen University, Shenzhen 518060, China; liuwj@szu.edu.cn (W.L.); xwbxwbxwb1996@gmail.com (W.X.)

[2] Fujian Key Laboratory of Functional Marine Sensing Materials, College of Materials and Chemical Engineering, Minjiang University, Fuzhou 350108, China

* Correspondence: gfdong@mju.edu.cn (G.D.); m.fang@szu.edu.cn (M.F.)

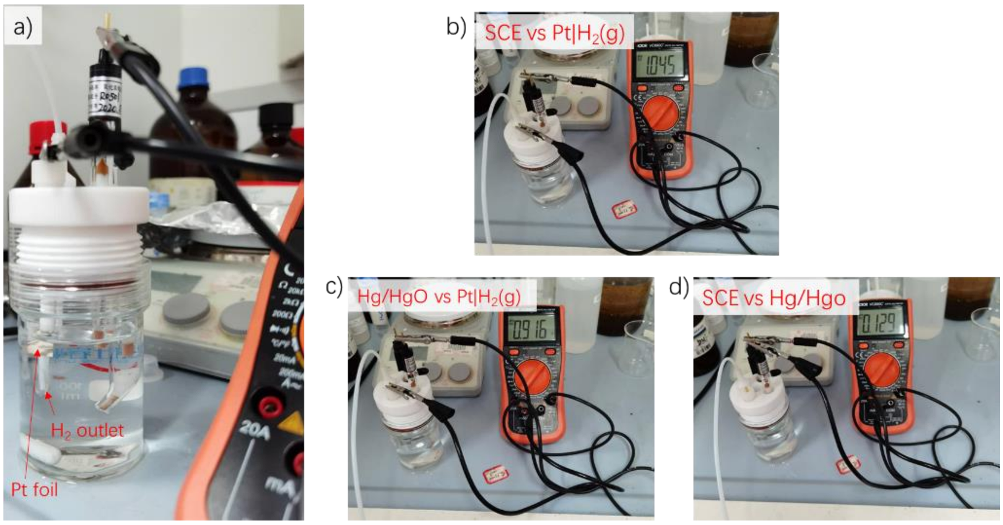

Figure S1. Potential calibration of the SCE and Hg/HgO against a Pt|H_2(g) electrode in 1 M KOH. Note: H_2 gas was bubbled against a fresh polished platinum foil for at least 30 min, and the voltage measurements were conducted immediately after shutting off the gas to avoid any mechanical disturbance by the gas bubbling.

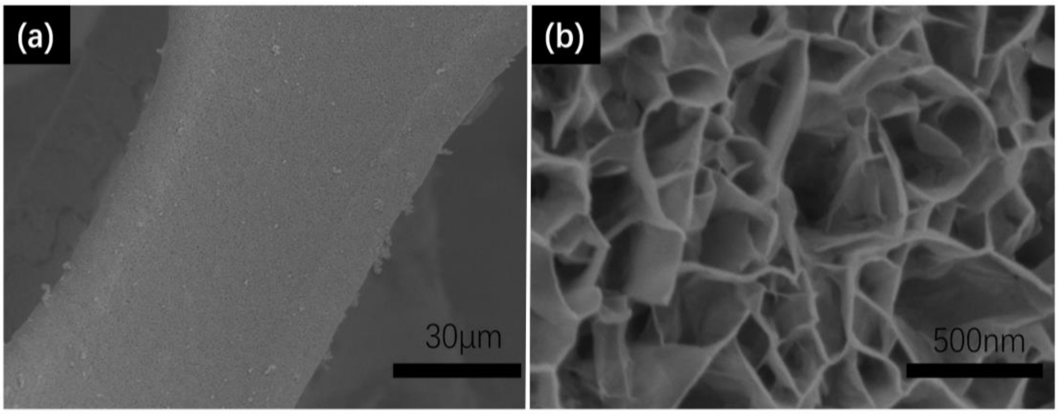

Figure S2. SEM images of MnO_2 nanosheet arrays grown on nickel foam (NF).

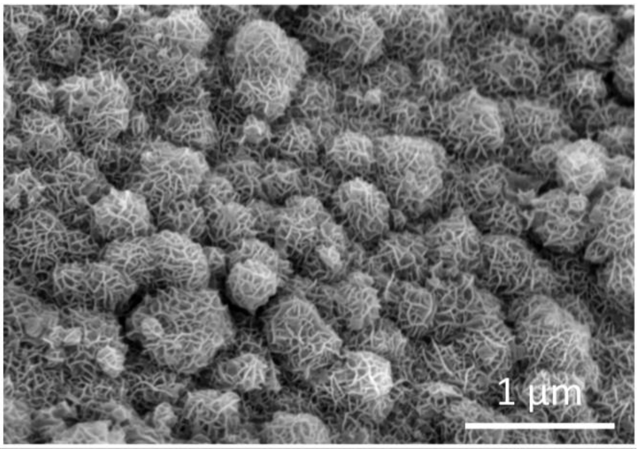

Figure S3. SEM image of CoNi-LDH deposited on NF.

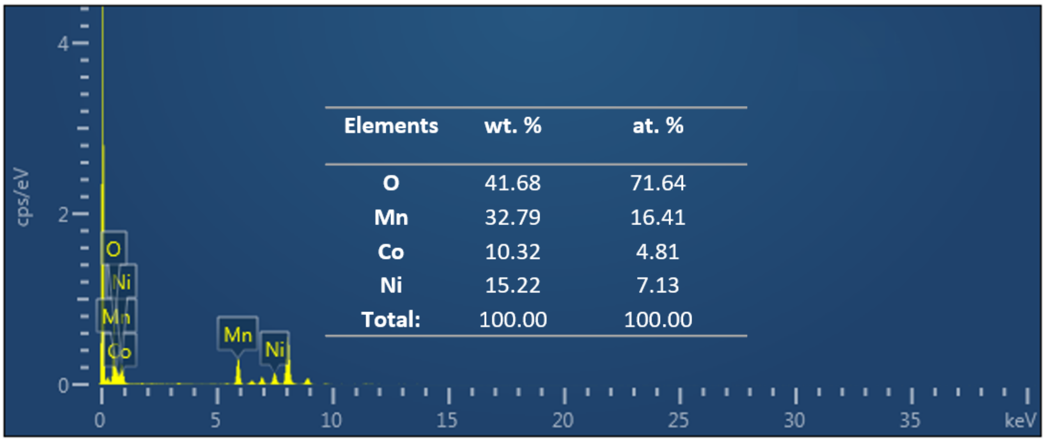

Figure S4. EDS spectrum of the mapping area.

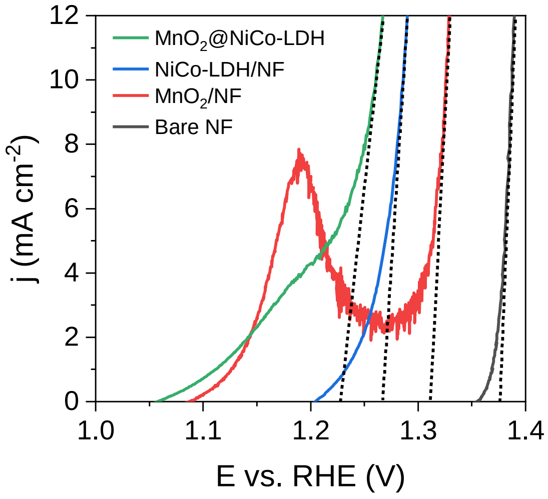

Figure S5. Enlarged view of the UOR LSV curves of different catalysts.

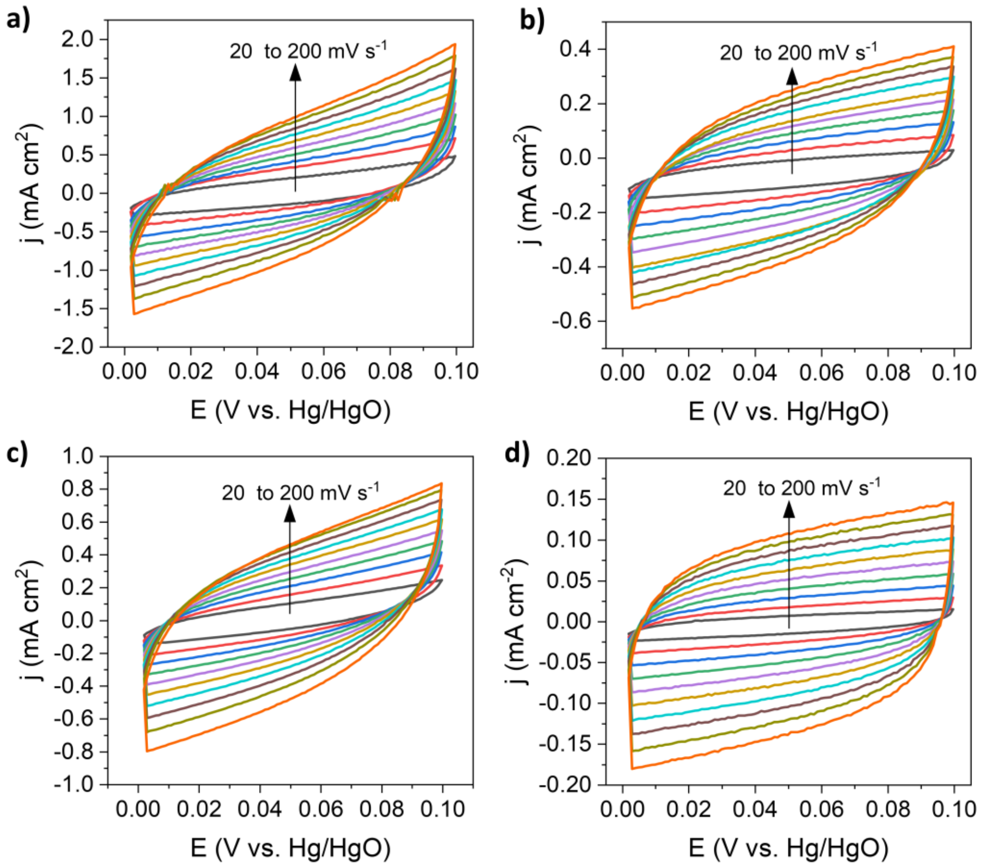

Figure S6. CV curves of a) MnO_2@NiCo-LDH, b) NiCo-LDH/NF, c) MnO_2/NF, and d) NF at different potential scan rates in the non-Faradaic potential range.

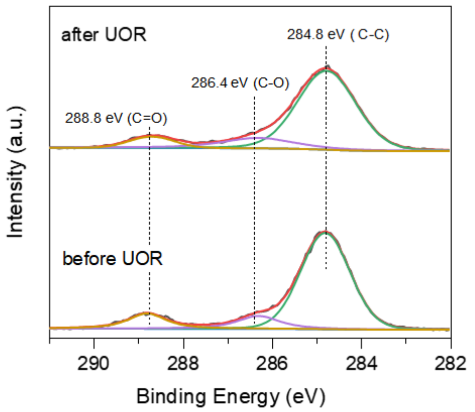

Figure S7: High-resolution calibrated C 1s XPS spectra of MnO_2@NiCo-LDH before and after the UOR test.

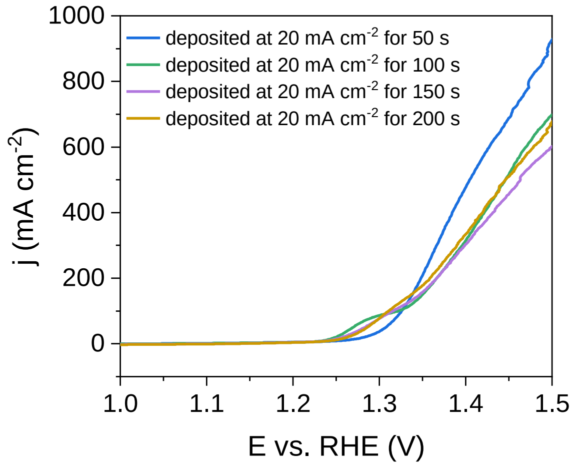

Figure S8. LSV curves of UOR of various MnO_2@NiCo-LDH samples with different deposition time.

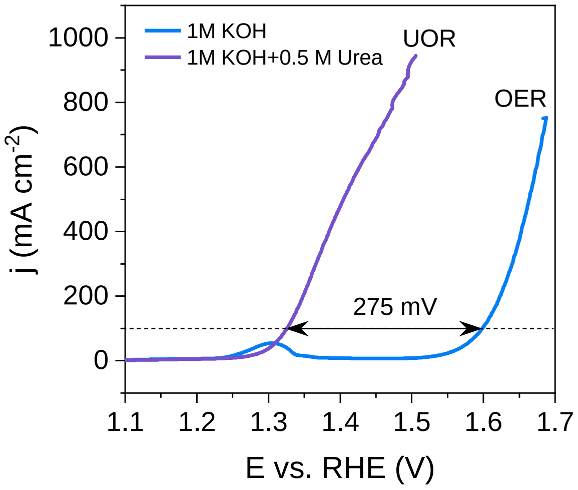

Figure S9. LSV curves of MnO_2@NiCo-LDH deposited for 50 s for UOR and OER.

Table S1. Performance comparison of UOR catalysts reported recently.

Catalysts	Electrolyte	*j* (mA cm^{-2})	Potential (V)	Reference
**CoS_2-MoS_2**	1.0 M KOH 0.5 M urea	10	1.29	Adv. Energy Mater., 2018, 1801775
**Ni_3N/NF**	1.0 M KOH 0.5 M urea	100	1.42	ACS Appl. Mater. Interfaces, 2019, 11, 13168
**Mo-Co-S-Se/CC**	1.0 M KOH 0.5 M urea	10	1.40	ACS Sustainable Chem. Eng., 2019, 7, 16577
**$NiMoO_3S$/NF**	1.0 M KOH 0.5 M urea	10	1.34	Chem. Commun., 2020, 56, 11038
**$NiSe_2$-NiO 350**	1.0 M KOH 0.33 M urea	10	1.33	Appl. Catal. B Environ., 2020, 276,119165
**CoFeCr LDH/NF**	1.0 M KOH 0.33 M urea	10	1.31	Appl. Catal. B Environ., 2020, 272,118959.
**$NiMo_xO_y$/NF**	1.0 M KOH 0.5 M urea	100	1.36	Applied Catalysis A, General, 2021 622, 118220
**$Ni_2Fe(CN)_6$**	1.0 M KOH 0.33 M urea	100	1.35	Nature Energy, 2021, 6, 904–912
**NiCoGe oxyhydroxide**	1.0 M KOH 0.33 M urea	100	1.33	Adv. Funct. Mater., 2023, 33, 2300687
**Ni-TPA@NiSe/NF**	1.0 M KOH 0.5 M urea	100	1.37	ACS Catal. 2023, 13, 837−847
**MnO_2@NiCo -LDH**	**1.0 M KOH 0.5 M urea**	**10**<br>**100**	**1.262**<br>**1.326**	**This work**
